# Preventive Strategies and Determinants of Vaccination Compliance Among Older Adults in Rural Amazonian Communities

**DOI:** 10.3390/vaccines14070643

**Published:** 2026-07-22

**Authors:** Luciana T. Pasquel-Muñoz, Ana Lucía Ramírez-Béjar, Joaquín Eduardo Chávez Cano, Kiara Camacho-Caballero, José F. Parodi, Fernando M. Runzer-Colmenares

**Affiliations:** 1 Carrera de Medicina Humana, Universidad Científica del Sur, Lima 15842, Peru; 100082257@cientifica.edu.pe (L.T.P.-M.); 100088657@cientifica.edu.pe (A.L.R.-B.); 100088623@cientifica.edu.pe (J.E.C.C.); 2 Sociedad Científica de Estudiantes de Medicina de la Universidad Científica del Sur, Lima 15842, Peru; 3 CHANGE Research Working Group, Carrera de Medicina Humana, Universidad Científica del Sur, Lima 15842, Peru; kcamacho@cientifica.edu.pe; 4 Internal Medicine, Naples Comprehensive Health (NCH) Healthcare System, Naples, FL 34102, USA; 5 Centro de Investigación del Envejecimiento, Facultad de Medicina Humana, Universidad de San Martín de Porres, Lima 15024, Peru; jparodig@usmp.pe

**Keywords:** older adults, vaccination, respiratory infections, frailty, Peruvian Amazon, rural health, immunization

## Abstract

Background/Objectives: Respiratory infections are a major cause of morbidity and mortality among older adults. However, vaccination coverage against respiratory pathogens remains suboptimal, particularly in rural and vulnerable populations. This study aimed to identify factors associated with respiratory vaccination uptake among older adults living in rural Amazonian communities in Peru. Methods: We conducted an observational, analytical, cross-sectional study using secondary data from the Amazon Frail Project. A total of 429 adults aged ≥60 years from rural and suburban communities in San Martín, Loreto, and Ucayali were included. Vaccination coverage for influenza, pneumococcal disease, pertussis, and COVID-19, as well as vaccine combinations, was assessed. Multiple linear and Poisson regression models with robust variance were used to identify associated factors. Results: Complete vaccination coverage was 42.89% for influenza, 23.08% for pneumococcal disease, 4.43% for pertussis, and 70.16% for COVID-19. Only 3.50% of participants had received all four respiratory vaccines. Higher educational attainment, multimorbidity, social frailty, greater muscle mass, better physical performance, and healthcare center attendance were associated with higher vaccination uptake. Depressive symptoms, cognitive impairment, functional dependency, physical frailty, dynapenia, and higher body mass index were associated with lower vaccination uptake. Conclusions: Respiratory vaccination coverage was suboptimal among older adults living in rural Amazonian communities. Educational, functional, physical, social, and healthcare access factors significantly influenced vaccine uptake, highlighting the need for targeted immunization strategies in vulnerable rural populations.

## 1. Introduction

During the aging process, the immune system response declines, making older adults (OA), people aged 60 years or older, more vulnerable to infections and complications that are often preventable through vaccination [1,2]. As the OA population continues to grow, projected to increase from 12.5% to 22–25% by 2050, low vaccination rates among this population represent an important public health concern [3]. Considerable worldwide disparities have been reported in vaccination coverage among older adults, with some countries exhibiting coverage rates below 25% for influenza and pneumococcal vaccines [4].

In Peru, where older adults account for 14.6% of the population, vaccination against respiratory diseases in this age group is implemented through the current National Vaccination Schedule, in accordance with the guidelines established by the Peruvian Ministry of Health [5]. This schedule applies nationwide, including in rural Amazonian communities. Compliance with this regulatory framework is mandatory for public healthcare facilities that provide immunization services; however, vaccination against infectious respiratory diseases among older adults is established as a prioritized national recommendation. Furthermore, vaccines are administered free of charge through immunization activities conducted at public healthcare facilities and during vaccination campaigns. These vaccines may also be obtained from private healthcare providers; nevertheless, the public immunization system remains the primary means of access [6].

Regarding the vaccines evaluated in this study, national recommendations include annual influenza vaccination, at least one dose of a pneumococcal vaccine, and COVID-19 vaccination in accordance with current regulations. Protection against pertussis was assessed through combined booster vaccines containing a pertussis component, such as Tdap [6]. The influenza vaccine is an inactivated seasonal influenza vaccine, whereas the pneumococcal vaccine is a conjugate polysaccharide vaccine. The COVID-19 vaccine is based on messenger RNA technology, while the combined pertussis vaccine (Tdap) contains tetanus and diphtheria toxoids together with acellular pertussis antigens [6,7].

Vaccination coverage among older adults in Peru has been estimated to vary considerably. Regarding pneumococcal vaccination, the Ministry of Health reported that 66.67% of older adults had been vaccinated in 2025, whereas a community-based study conducted in selected Peruvian cities found that only 27% of older adults had received influenza vaccination during the previous year [8,9]. During the COVID-19 pandemic in Peru, older adults accounted for 68.8% of confirmed deaths, of which 96.4% were attributed to COVID-19-related pneumonia. In rural Amazonian communities, such as Condorcanqui in the Amazonas region, a cumulative incidence of 63.84 cases per 1000 inhabitants was reported; of these, 308 cases occurred among individuals aged 60 years or older, representing 9.4% of all cases [10,11]. Regarding pneumonia, one report indicated that older adults accounted for 75.6% of deaths due to pneumonia of unspecified etiology, suggesting potential limitations in etiological diagnosis [12].

The high prevalence of preventable respiratory diseases and vaccines in specific regions, such as the northern Peruvian Amazon, may reflect existing disparities in annual vaccination efforts geared specially towards this high risk population by the Ministry of Health, reflecting substantial disparities in health status, access to healthcare services, and vaccination coverage compared with other vulnerable populations in Peru, including urban communities living in poverty and other geographically isolated populations, largely driven by limited healthcare infrastructure, shortages of healthcare personnel, and sociocultural barriers to healthcare access [13].

Several factors have been associated with non-vaccination among OA, including poor mental health, depressive symptoms, and dementia [14,15]. Multimorbidity has also been associated with higher vaccination rates because these individuals are often prioritized during public health campaigns [16]. Lower education levels and socioeconomic status have also been associated with lower vaccination uptake [17,18]. In contrast, the relationship between frailty and vaccination remains inconsistent. Although some studies suggest that frailty is associated with higher vaccination rates, others have reported opposite findings [19]. Furthermore, evidence on these associations in rural settings remains limited.

Given these gaps, this study aimed to evaluate the factors associated with respiratory vaccination adherence among OA living in rural Amazonian communities in northern Peru.

## 2. Methods

This was an observational, analytical, cross-sectional study based on a secondary analysis of data from the Amazon Frail Project. The original study was conducted between 2024 and 2025 in rural and suburban communities of the Peruvian Amazon, located in the regions of San Martín, Loreto, and Ucayali. These communities are characterized by land and/or river access, poverty or extreme poverty, and limited access to basic services and healthcare [20].

The original study included adults aged 60 years or older residing in the selected communities. Participants were excluded if they had eight or more errors on the Pfeiffer Short Portable Mental Status Questionnaire [21], visual impairment that prevented completion of physical performance tests, hearing impairment that limited participation in the interview, refusal to participate, or limitations in understanding or communicating in Spanish. A non-probability census sampling method was used. The primary objective of the original study was to identify characteristics associated with physical, mental, and social frailty among OA.

**Figure 1:**
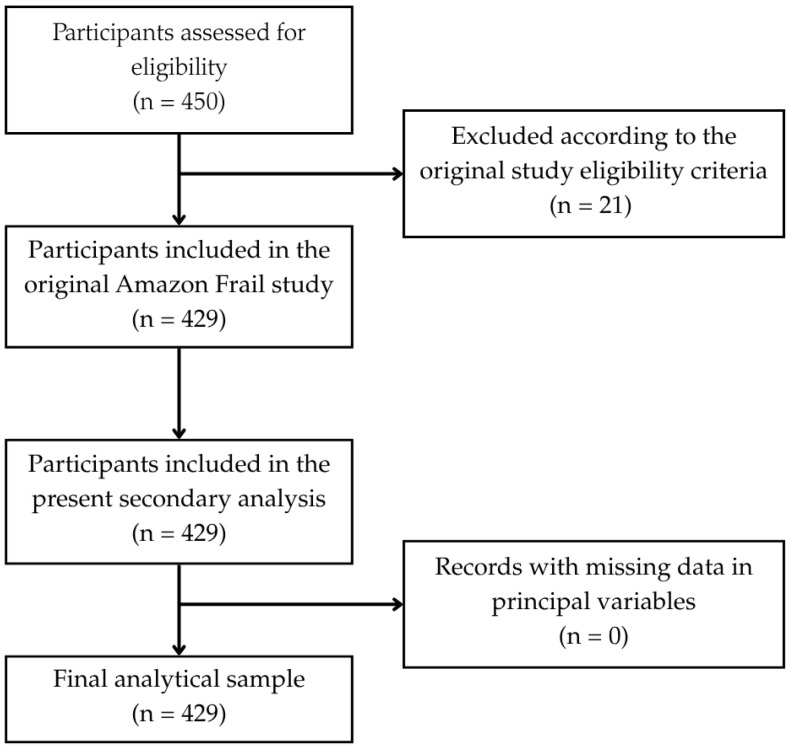
Flowchart of participant selection for secondary data analysis.

For this secondary analysis, all participants in the primary study were considered eligible. Records with missing data in the variables of interest were defined as an exclusion criterion; however, no incomplete records were identified for any principal variables. The final sample included 429 participants (Figure 1).

### 2.1. Variables

The outcome of interest was adherence to the vaccination schedule, assessed using a questionnaire item that involved direct verification of the participant’s vaccination card [2]. The vaccines evaluated were influenza, pneumococcal, COVID-19, and pertussis vaccines. For the statistical analysis, the number of respiratory vaccines received by each participant was analyzed as a numerical variable. In addition, exploratory variables were created based on combinations of the available respiratory vaccines (influenza plus pneumococcal; Influenza plus pertussis; influenza + COVID-19; pneumococcal + COVID-19; pneumococcal + pertussis; COVID-19 + pertussis) [2].

Covariates were grouped as sociodemographic, including age (60–79 and ≥80 years), sex (male and female), marital status (single, married/cohabiting, and divorced/separated), and educational level (illiterate/incomplete primary education; complete primary and secondary education; and higher technical education).

Clinical characteristics included multimorbidity, functional dependence, frailty, malnutrition and depressive symptoms. Multimorbidity was defined as the presence of two or more medical conditions, including hepatitis B, asthma, myocardial infarction, malaria, hypertension, chronic kidney disease, dengue, congestive heart failure, COVID-19, stroke, leishmaniasis, type 2 diabetes mellitus, chronic obstructive pulmonary disease, among others [22]. Functional dependence in basic activities of daily living was assessed using the Barthel index, with scores below 100 indicating dependence [23]. Functional dependence in instrumental activities of daily living was evaluated using the Lawton Index, with cutoff points of ≤7 for women and ≤4 for men [24]. Frailty was assessed using the FRAIL scale, which assesses fatigue, resistance, ambulation, illnesses, and weight loss, and was subsequently classified into three categories: non-frail (0 points), pre-frail (1 point), and frail (≥2 points) [25]. Malnutrition was assessed using the Mini Nutritional Assessment-Elderly, with a cutoff score of 23.5 or lower considered positive according to the Integrated Care for Older People (ICOPE) manual proposed by the Pan American Health Organization (PAHO) [26]. Depressive symptoms were evaluated using the 5-item Yesavage Geriatric Depression Scale, with a score of two or more points considered positive [27]. Social frailty was assessed using the Social Frailty Index (SFI), which consists of 10 items including social interaction, community participation, perception of the environment, and economic situation. The SFI score was obtained by calculating the log-odds (*lp*) of the scores for each of the 10 items using the following formula: *lp* = −9.210517 + 0.112097 × *age* − 0.553411 × *gender* + 0.497577 × *meetup*_*child* + 0.107782 × *activity*1 + 0.48419 × *activity*2 − 0.255202 × *isolate* + 0.351463 × *area* − 0.438733 × *finance* − 0.291638 × *treat* − 0.553316 × *work*. Subsequently, the lp scores were transformed into a probability using the logistic function: *pr* = *e* *lp* 1 + *e* *lp*/(1 + *e* *lp*) × 100. Finally, the total score was transformed into tertiles: tertile 1 (lower risk of frailty), tertile 2 (intermediate frailty) and tertile 3 (higher frailty) [20].

Finally, a third category of covariates included anthropometric and body composition variables. Body Mass Index (BMI) was calculated as weight (kg)/height (m)^2^, with values ≤ 18.4 kg/m^2^ classified as underweight, 18.5–24.9 kg/m^2^ as normal weight, 25.0–29.9 kg/m^2^ as overweight, and ≥30 kg/m^2^ as obesity [28]. The Skeletal Muscle Mass Index, measured through bioelectrical impedance analysis (SMI = appendicular muscle mass/height^2^), was used to assess muscle mass, with low muscle mass defined as ≤7.0 kg/m^2^ in men and ≤5.7 kg/m^2^ in women [29]. The waist-hip ratio (WHR) was calculated as waist circumference/hip circumference, with values > 1.0 in men and >0.85 in women considered indicative of increased health risk [30].

### 2.2. Statistical Analysis

Statistical analysis was performed using STATA version 18. Categorical variables were summarized as frequencies and percentages. Numerical variables were summarized as frequencies and percentages or means and standard deviations, since their distributions were considered approximately normal on histogram inspection.

Multiple linear regression models were conducted to estimate β coefficients, robust standard errors, *p*-values, and 95% confidence intervals (95% CIs). In these models, the number of respiratory vaccines received was treated as a continuous dependent variable. Age and sex were not included as covariates because they are confounders of the definition of other variables, such as muscle mass and muscle strength, in order to avoid model over-adjustment.

In the present study, a linear regression model was used to estimate the beta coefficients associated with the number of vaccines received (a discrete count variable ranging from 0 to 4), treating it as an ordinal quantitative variable because its empirical distribution and behavior allowed it to be reasonably approximated as a continuous scale while preserving the clinical interpretability of the marginal effects. In this context, the beta coefficients are interpreted as the mean change in the number of vaccines associated with a one-unit increase in each covariate, under the assumptions of linearity, homoscedasticity, and approximate normality of the residuals, all of which were verified. The use of linear regression, rather than count-specific models, is further justified by the bounded range and low dispersion of the outcome, which support stable parameter estimates and facilitate a more transparent and clinically interpretable presentation of the findings, consistent with the recommendations of contemporary biostatistical literature applied to clinical and epidemiological research.

Before performing the linear regression, the statistical assumptions were assessed using the Shapiro–Wilk test for normality, White’s test to evaluate heteroscedasticity, and a Q–Q plot to examine the normality of the residuals.

In addition, multiple Poisson regression models with robust variance were constructed to estimate prevalence ratios (PRs) and their corresponding 95% confidence intervals. Six models were developed, each considering a different combination of respiratory vaccines as the dependent variable. For both linear and Poisson regression models, statistical assumptions were evaluated, including independence, linearity, and Poisson distribution assumptions. Multicollinearity post hoc evaluations were also performed through the calculation of variance inflation factors (VIFs). No variables were excluded on the basis of multicollinearity, as all VIF values were below 2. It should be noted that, for Models 5 and 6, the body mass index (BMI) variable was excluded due to insufficient sample size.

Regarding statistical power, calculations were performed using OpenEpi 3.0 software, assuming a 95% confidence level and a sample size of 429 participants. Statistical power was estimated under four scenarios based on functional status, multimorbidity, marital status, and educational level. Complete vaccination frequencies reported in previous studies were used for each comparison group. In all four scenarios, the estimated statistical power exceeded 80%, indicating that the sample size was adequate for the analysis of the groups of interest.

### 2.3. Ethical Considerations

The research project was approved by the Institutional Research Ethics Committee of Universidad Científica del Sur. All participants were recruited after providing their written informed consent. In cases where cognitive impairment was identified, informed consent was obtained from the legally authorized representative or accompanying caregiver of the participant.

The database used for this analysis was fully anonymized and contained no information that could identify individual participants. The original project was funded by the Vice-Rectorate for Research of Universidad Científica del Sur through the Semilla 2023 Research Grant Program. Additional funding was provided by the Institute of Human Medicine of Universidad San Martín de Porres.

## 3. Results

Table 1 presents the main characteristics of the study population. Most participants were between 60 and 79 years old, and 64.02% (n = 274) were female. Regarding individual vaccines, 42.89% (n = 184) had received influenza vaccination, 23.08% (n = 99) pneumococcal vaccination, 4.43% (n = 19) pertussis vaccination, and 70.16% (n = 301) COVID-19 vaccination.

For combined vaccine schedules, 22.14% (n = 95) had completed the influenza—pneumococcal schedule, 4.43% (n = 19) had completed the influenza-pertussis schedule, 32.87% (n = 141) had completed the influenza -COVID-19 schedule, and 18.41% (n = 79) had completed the pneumococcal-COVID-19 schedule. Furthermore, combinations of pneumococcal-pertussis and COVID-19-pertussis vaccines were observed in 3.50% of participants for each schedule (n = 15 in both cases). Finally, regarding the number of respiratory vaccines received per participant, only 13.99% (n = 60) had received at least three of the four recommended respiratory vaccines, while 3.50% (n = 15) had completed the full four-vaccine schedule. Further details are presented in Table 1.

Table 2 shows the descriptive analysis of the numerical variables included in the study. The mean age of the participants was 70.46 years (standard deviation [SD]: 7.60). In addition, participants received a mean of 1.41 respiratory vaccines (SD: 1.06). More details are presented in Table 2.

Table 3 shows the multiple linear regression analysis evaluating the association between covariates and the number of respiratory vaccines received per participant (n = 429). Participants with school education had a higher mean number of respiratory vaccines received compared with illiterate participants (β = 0.30; 95% CI: 0.08–0.52). However, higher scores on the Yesavage Geriatric Depression Scale were associated with fewer respiratory vaccines received (β = −0.14; 95% CI: −0.20 to −0.07).

Higher Barthel Index scores, representing greater independence in basic activities of daily living, were associated with a lower mean number of respiratory vaccines received (β = −0.01; 95% CI: −0.02 to −0.01). Social frailty was associated with an increased mean number of respiratory vaccines received. Compared with participants with low social frailty, those with moderate social frailty received a significantly higher mean number of vaccines (β = 0.58; 95% CI: 0.35–0.81), and this association was even stronger among participants with high social frailty (β = 0.67; 95% CI: 0.38–0.96). In relation to muscle mass, greater muscle mass, measured by bioelectrical impedance analysis, was associated with a higher mean number of respiratory vaccines received (β = 0.16; 95% CI: 0.07–0.26). Similarly, participants with higher Short Physical Performance Battery (SPPB) scores received a greater mean number of respiratory vaccines (β = 0.05; 95% CI: 0.01–0.09). Participants with multimorbidity also received a significantly higher mean number of respiratory vaccines than those without multimorbidity (β = 0.36; 95% CI: 0.12–0.59). Finally, higher body mass index (BMI) was associated with a lower mean number of respiratory vaccines received (β = −0.03; 95% CI: −0.06 to −0.01). Further details are presented in Table 3.

Table 4 presents the adjusted Poisson regression analysis performed to quantify the association between covariates and respiratory vaccine combinations in the study population (n = 429). Six models were constructed based on the following vaccine combinations: influenza plus pneumococcal (Model 1), influenza plus pertussis (Model 2), influenza plus COVID-19 (Model 3), pneumococcal plus COVID-19 (Model 4), pneumococcal plus pertussis (Model 5), and COVID-19 plus pertussis (Model 6).

Overall, higher educational level was associated with a greater likelihood of receiving the influenza plus pertussis, influenza plus COVID-19, pneumococcal plus pertussis and COVID-19 plus pertussis vaccine combinations. Conversely, cognitive impairment and depressive symptoms were associated with a lower likelihood of receiving the influenza plus pneumococcal vaccine combination. Functional dependency was negatively associated with the influenza plus pertussis and pneumococcal plus COVID-19 combinations, while physical frailty and dynapenia were associated with a lower likelihood of receiving the pneumococcal plus pertussis and COVID-19 plus pertussis combinations. Participants with moderate or high social frailty were more likely to receive the influenza plus pneumococcal vaccine combination, whereas high social frailty was additionally associated with greater uptake of the influenza plus pertussis, influenza plus COVID-19 and pneumococcal plus COVID-19 combinations. Low muscle mass was associated with a lower likelihood of receiving the influenza plus pneumococcal and influenza plus COVID-19 combinations. Finally, participants who attended health centers had a greater likelihood of receiving the influenza plus pneumococcal, influenza plus COVID-19 and pneumococcal plus COVID-19 vaccine combinations. More details are presented in Table 4.

## 4. Discussion

Our study evaluated the factors and determinants associated with adherence to the respiratory immunization schedule among older adults living in rural Amazonian communities. Overall, our findings indicate that vaccination coverage in this group was suboptimal and was associated with factors from different categories, including sociodemographic factors, certain geriatric syndromes, and use of health services. Overall, these findings suggest that adherence to the immunization schedule in this group should be interpreted from an integral geriatric perspective, considering individual characteristics of older adults that may limit or facilitate access to vaccination and should be identified in a timely manner during contact with health services.

Educational level was significantly associated with the probability of receiving respiratory vaccines among older adults. In particular, older adults who were illiterate received a lower number of respiratory vaccines, whereas those with a higher educational level were more likely to receive them. This association may suggest that a higher educational level is related to a better understanding of health-related instructions, as well as deeper knowledge about vaccination and its benefits [31]. This is consistent with the literature, where low educational level has been associated with lower health literacy, understood as a reduced ability to understand, interpret, and use health information for decision-making [32]. This may limit the understanding of medical instructions, the identification of which vaccines correspond to each individual, especially when multiple immunization schedules are involved, and the appropriate follow-up of indicated doses [15]. Thus, these findings highlight the need to adapt vaccination communication strategies for older adults with low educational level, using clear and concise messages.

On the other hand, individuals with mental health problems, such as depressive symptoms or memory loss, were found to have a lower probability of receiving the influenza and pneumococcal vaccine combination. Several studies have shown that the presence of concomitant mental health problems in the older adult population is associated with reduced access to healthcare interventions such as vaccination [33,34]. This occurs because these mental health problems can significantly hinder understanding of medical instructions, as well as the ability to remember them over time and maintain the motivation to seek medical care proactively [35]. Similarly, among individuals with memory loss, intrapersonal barriers, such as cognitive impairment; interpersonal barriers related to the role of caregivers and the burden they may experience; and extrapersonal barriers, such as social isolation, have been described [15]. In this sense, these findings highlight the importance of reorienting the current approach to vaccination campaigns for older adults. Therefore, these findings suggest that depressive symptoms and cognitive impairment should also be considered when assessing vaccination in older adults.

Regarding functional status, multimorbidity, and body mass index (BMI), the associations differed according to the vaccine outcome evaluated. Functional dependency was associated with lower uptake of vaccine combinations, likely because reduced mobility, transportation barriers, and dependence on caregivers limit access to vaccination services [36,37]. In contrast, multimorbidity was associated with a higher probability of receiving respiratory vaccines, which is consistent with immunization strategies that prioritize individuals with chronic diseases and with previous evidence showing that greater disease burden increases opportunities for healthcare providers to recommend and administer vaccines during routine medical visits [38]. Regarding BMI, we found an inverse association with the number of respiratory vaccines received, where higher BMI was associated with lower vaccine uptake. This association should be interpreted with caution, since this finding differs from previous studies that reported higher vaccination rates among individuals with obesity, possibly due to more frequent healthcare utilization and recognition of obesity as a metabolic risk factor [39].

Social frailty was associated with a higher probability of receiving respiratory vaccines, indicating that sociofamilial protective factors and social networks are relevant and are adequately recognized as such by the Peruvian health system as part of vaccination campaign strategies and the identification of older adults who are vulnerable in this regard. Similarly, perceived social support and support have been described as factors that facilitate timely access to respiratory vaccines [40]. In contrast, with regard to physical and muscular frailty, a lower uptake of some combinations of respiratory vaccines was observed, suggesting a gap in muscle health screening, specifically for sarcopenia, particularly in these rural settings [41]. However, people with adequate physical performance had a higher probability of being vaccinated, especially with the influenza–pneumococcal combination, suggesting that adequate muscle health can promote independence and mobility to attend health centers.

In the use of health services, people who attended primary healthcare centers when medical care was needed were found to have greater coverage of respiratory vaccination. This finding emphasizes the importance of primary healthcare centers in identifying older adults and increasing vaccination opportunities, regardless of the initial reason for consultation [42]. These results are consistent with the literature, which describes that the use of health services and affiliation with a health network increase the likelihood of vaccination by facilitating counseling and access to information [43].

Our findings provide strong empirical evidence for more explicitly integrating vaccination into person-centered care models, such as the WHO’s ICOPE and the development of age-friendly services, at the micro, meso, and macro levels [44]. At the individual (micro) level, each older adult should be assessed according to their clinical, functional, and social circumstances. To this end, primary care protocols should include, from the beginning of each consultation and regardless of the reason for attendance, a review of the vaccination card, documentation of administered and pending doses, and application of the ICOPE screening tool by appropriately trained healthcare personnel. These measures would help bring overdue vaccinations up to date, reduce missed opportunities for vaccination in this age group, and facilitate the identification of vulnerable older adults. In this context, the coexistence of multimorbidity, impaired intrinsic capacity, physical and social frailty, and very low respiratory vaccination coverage among older adults already in contact with primary care highlights the need to incorporate routine assessment of vaccination status into ICOPE screening and individualized care plans, recognizing vaccination as a core component of preserving intrinsic capacity, rather than optional add-ons [44].

At the service level (meso), the significant gap between frequent use of health centers and missed vaccination opportunities suggests that services aligned with the 5Ms framework and age-friendly services should redesign operational workflows, protocols, and team roles so that each encounter with a frail older person triggers proactive and personalized vaccination offers, along with medication review, mobility support, and mental status assessment. The approach should not be limited to older adults with chronic conditions, such as cardiovascular or metabolic diseases, but should also include those with geriatric syndromes and barriers to healthcare access. Healthcare personnel should also assume an active role in providing clear, understandable, and age-friendly vaccination guidance. This role could be strengthened through periodic training in communication strategies tailored to each older adult’s sociocultural context and cognitive status. Furthermore, to reach the target population, healthcare centers should support the deployment of mobile land- and river-based vaccination teams to hard-to-reach communities, as well as regular home visits for older adults identified as having geriatric syndromes. These interventions should also seek to strengthen support networks, including caregivers, family members, and community health workers, by communicating vaccination dates and facilitating follow-up [45,46].

At the policy level (macro), documenting this combination of high vulnerability and low vaccination coverage in a real-world primary care setting supports the inclusion of adult vaccination indicators in national ICOPE-oriented strategies. The number of completed vaccination schedules should be recorded periodically and disaggregated by region, vulnerability status, and healthcare facility. This incentivizes health systems to prioritize and fund preventive interventions for those with the greatest needs and to align funding and performance metrics with the 5Ms objective of providing what matters most to older people.

## 5. Limitations

This study had some limitations. First, the selection of communities and participants was based on a nonprobability sampling method, which may limit the generalizability of the findings. Additionally, due to the cross-sectional design, the associations observed between the factors studied and the outcome do not allow causal inferences to be established.

Furthermore, information on the number of specific cases of influenza, pneumococcal disease, pertussis, and COVID-19 was not available. Likewise, it would have been valuable to include serological measurements to assess immune status and confirm the level of protection among participants. The inclusion of these variables could have provided a more comprehensive answer to the research question.

In the study communities, COVID-19 vaccination may have been promoted through Pensión 65, a non-contributory social pension program for older adults living in extreme poverty. Although this information was not systematically recorded, it may partially explain the relatively high COVID-19 vaccination coverage observed in our population and should be considered a potential unmeasured confounding factor.

Additionally, the exclusion of older adults with advanced dementia may have resulted in an overestimation of vaccination uptake in our cohort, as it excluded a subgroup that likely experiences a greater burden of cognitive, functional and logistical barriers to accessing healthcare services, attending scheduled appointments or maintaining vaccination records. However, in some rural settings, home-based vaccination strategies are available and may partially mitigate these limitations; therefore, the magnitude and direction of the potential bias introduced by our eligibility criteria cannot be determined with certainty. In this regard, it is important to emphasize that vaccination among individuals with major neurocognitive disorders requires specific and tailored methodologies, including systematic home-based vaccination strategies, close coordination with caregivers and simplified record-keeping systems. These considerations were beyond the scope of the present study but should be addressed in future research and considered when interpreting our findings.

Despite these limitations, the study provides relevant and novel evidence from a population rarely studied. Furthermore, data were collected by trained healthcare professionals using validated instruments, which improves the quality and reliability of the information obtained. In this sense, the findings contribute to a better understanding of the factors associated with adherence to the vaccination schedule among older adults living in rural Amazonian communities.

## 6. Conclusions

This study identified multiple factors associated with the coverage of respiratory vaccination among older adults living in rural Amazonian communities, in a context characterized by suboptimal uptake of vaccines. The findings suggest that vaccination strategies in rural Amazonian settings should move beyond disease-based prioritization and incorporate the active identification of geriatric syndromes, functional limitations and barriers to healthcare access. In addition to this, to reduce the coverage gaps identified among geriatric populations in Amazonian settings, it is necessary to strengthen support networks for vulnerable older adults and increase opportunities for vaccination and counseling during contact with primary healthcare centers and community spaces.

## Figures and Tables

**Table 1:** Descriptive analysis of the main variables of the study (n = 429).

Characteristics	n	%
Age in years		
60–79	368	85.78
80 and over	61	14.22
Sex		
Female	274	64.02
Male	154	35.98
Level of education		
Illiterate	114	26.70
Incomplete primary/Complete primary/Incomplete secondary	293	68.62
Complete secondary/Higher technical education	20	4.68
Pfeiffer Questionnaire score		
No	342	79.72
Yes	87	20.28
5-item Yesavage Questionnaire score		
No	268	62.62
Yes	160	37.38
Barthel Index score		
No	286	67.14
Yes	140	32.86
Social frailty		
Low	144	33.57
Medium	143	33.33
High	142	33.10
Number of frailty criteria according to Fried phenotype		
Robust	316	73.66
Fragile	113	26.34
Muscle mass according to bioimpedance (kg)		
Normal	263	61.45
Low	165	38.55
Handgrip strength (kg)		
Normal	208	48.48
Weak	221	51.52
Short Physical Performance Battery score		
Normal	291	67.83
Abnormal	138	32.17
Multimorbidity		
No	116	27.04
Yes	313	72.96
Body Mass Index (kg/m^2^)		
Underweight	22	5.13
Normal	148	34.50
Overweight	152	35.43
Obese	107	24.94
Where to seek medical attention		
Self-medication, pharmacy, traditional medicine, and other	165	38.46
Health center	264	61.54
Influenza vaccination		
Incomplete	245	57.11
Complete	184	42.89
Pneumococcal vaccination		
Incomplete	330	76.92
Complete	99	23.08
Pertussis vaccination		
Incomplete	410	95.57
Complete	19	4.43
COVID-19 vaccination		
Incomplete	128	29.84
Complete	301	70.16
Combined Influenza + Pneumococcal vaccination schedule		
Incomplete	334	77.86
Complete	95	22.14
Combined Influenza + Pertussis vaccination schedule		
Incomplete	410	95.57
Complete	19	4.43
Combined Influenza + COVID-19 vaccination schedule		
Incomplete	288	67.13
Complete	141	32.87
Combined Pneumococcal + COVID-19 vaccination schedule		
Incomplete	350	81.59
Complete	79	18.41
Combined Pneumococcal + Pertussis vaccination schedule		
Incomplete	414	96.50
Complete	15	3.50
Combined COVID-19 + Pertussis		
Incomplete	414	96.50
Complete	15	3.50
Number of respiratory vaccines per participant		
None	85	19.81
1	175	40.79
2	94	21.91
3	60	13.99
Complete schedule	15	3.50

For the descriptive analysis, some variables might not add up to the final sample size, but in no case did the missing data exceed 10%.

**Table 2:** Descriptive analysis of the numerical variables (n = 429).

Variables	Average	Standard Deviation
Age in years	70.46	7.60
Pfeiffer Questionnaire score	1.74	1.90
5-item Yessavage Questionnaire score	1.41	1.63
Barthel Index score	90.15	12.57
Number of frailty criteria according to Fried phenotype	2.00	1.00
Muscle mass according to bioimpedance (kg)	5.56	1.43
Handgrip strength (kg)	19.27	6.31
Short Physical Performance Battery score	9.31	2.93
Body mass index (kg/m^2^)	26.57	4.84
Number of respiratory vaccines per participant	1.41	1.06

**Table 3:** Multiple linear regression analysis to quantify factors associated with the number of respiratory vaccines per participant (n = 429).

Variables	Beta Coefficient	Robust Standard Error	*p*-Value	(95% Confidence Interval)
Level of education				
Illiterate	Reference	Reference	Reference	Reference
Incomplete primary/Complete primary/Incomplete secondary	0.30	0.11	0.007	(0.08 to 0.52)
Complete secondary/Higher technical education	0.31	0.26	0.226	(−0.19 to 0.81)
Pfeiffer Questionnaire score	−0.03	0.03	0.334	(−0.09 to 0.03)
5-item Yesavage Questionnaire score	−0.14	0.03	0.001	(−0.20 to −0.07)
Barthel Index score	−0.01	0.01	0.008	(−0.02 to −0.01)
Social frailty				
Low	Reference	Reference	Reference	Reference
Medium	0.58	0.12	0.001	(0.35 to 0.81)
High	0.67	0.15	0.001	(0.38 to 0.96)
Number of frailty criteria according to Fried phenotype	0.03	0.06	0.647	(−0.09 to 0.14)
Muscle mass according to bioimpedance (kg)	0.16	0.05	0.001	(0.07 to 0.26)
Handgrip strength (kg)	0.01	0.01	0.254	(−0.01 to 0.03)
Short Physical Performance Battery score	0.05	0.02	0.040	(0.01 to 0.09)
Multimorbidity				
No	Reference	Reference	Reference	Reference
Yes	0.36	0.12	0.003	(0.12 to 0.59)
Body mass index (kg/m^2^)	−0.03	0.01	0.030	(−0.06 to −0.01)
Where to seek medical attention				
Self-medication, pharmacy, traditional medicine, and others	Reference	Reference	Reference	Reference
Health center	0.20	0.10	0.060	(−0.01 to 0.40)
Pseudo R2 = 0.017				

**Table 4:** Adjusted Poisson Regression Analysis to determine factors associated with combination of respiratory vaccines (n = 429).

Variables	Model 1: Influenza + Pneumococcal	Model 2: Influenza + Pertussis	Model 3: Influenza + COVID-19	Model 4: Pneumococcal + COVID-19	Model 5: Pneumococcal + Pertussis	Model 6: COVID-19 + Pertussis
	RP (95% Confidence Interval)	RP (95% Confidence Interval)	RP (95% Confidence Interval)	RP (95% Confidence Interval)	RP (95% Confidence Interval)	RP (95% Confidence Interval)
Level of education						
Illiterate	Reference	Reference	Reference	Reference	Reference	Reference
Incomplete primary/Complete primary/Incomplete secondary	1.13 (0.69–1.86)	3.31 (0.78–14.12)	1.67 (1.12–2.49)	1.79 (0.93–3.43)	1.47 (1.09–1.78)	1.14 (1.02–1.78)
Completed secondary/Higher technical education	1.72 (0.81–3.63)	1.03 (1.02–1.08)	1.92 (1.04–3.56)	2.24 (0.98–5.09)	1.99 (1.62–2.06)	1.92 (1.35–2.61)
Pfeiffer Questionnaire score						
No	Reference	Reference	Reference	Reference	Reference	Reference
Yes	0.46 (0.24–0.86)	0.32 (0.06–1.64)	0.72 (0.46–1.12)	0.47 (0.22–1.02)	0.64 (0.12–3.31)	0.64 (0.12–3.31)
5-item Yesavage Questionnaire score						
No	Reference	Reference	Reference	Reference	Reference	Reference
Yes	0.56 (0.37–0.87)	0.69 (0.32–1.49)	0.80 (0.59–1.08)	0.79 (0.52–1.22)	0.50 (0.14–1.80)	0.50 (0.14–1.80)
Barthel Index score						
No	Reference	Reference	Reference	Reference	Reference	Reference
Yes	0.77 (0.50–1.18)	0.41 (0.19–0.85)	1.12 (0.81–1.55)	0.59 (0.37–0.95)	0.47 (0.13–1.63)	0.47 (0.13–1.63)
Social frailty						
Low	Reference	Reference	Reference	Reference	Reference	Reference
Medium	1.90 (1.21–3.00)	1.07 (0.24–4.85)	1.31 (0.91–1.88)	1.52 (0.94–2.47)	0.35 (0.09–1.32)	0.35 (0.09–1.32)
High	2.97 (1.95–4.51)	2.84 (1.16–6.93)	1.64 (1.14–2.35)	2.83 (1.82–4.39)	2.13 (0.75–5.99)	2.13 (0.75–5.99)
Number of frailty criteria according to Fried phenotype						
Robust	Reference	Reference	Reference	Reference	Reference	Reference
Fragile	0.86 (0.50–1.45)	1.12 (0.93–1.57)	0.84 (0.57–1.24)	1.08 (0.55–2.09)	0.85 (0.76–0.98)	0.35 (0.26–0.78)
Muscle mass according to bioimpedance (kg)						
Normal	Reference	Reference	Reference	Reference	Reference	Reference
Low	0.56 (0.36–0.88)	0.61 (0.24–1.51)	0.66 (0.48–0.92)	0.60 (0.36–1.01)	0.60 (0.21–1.67)	0.60 (0.21–1.67)
Handgrip strength (kg)						
Normal	Reference	Reference	Reference	Reference	Reference	Reference
Weak	0.73 (0.48–1.12)	0.56 (0.17–1.81)	1.06 (0.76–1.48)	0.59 (0.34–1.03)	0.21 (0.06–0.72)	0.21 (0.06–0.72)
Short Physical Performance Battery score						
Normal	Reference	Reference	Reference	Reference	Reference	Reference
Abnormal	1.66 (1.10–2.51)	1.16 (0.24–5.64)	0.95 (0.67–1.35)	0.97 (0.60–1.57)	0.35 (0.11–1.09)	0.35 (0.11–1.09)
Multimorbidity						
No	Reference	Reference	Reference	Reference	Reference	Reference
Yes	1.44 (0.86–2.42)	3.15 (0.74–13.45)	1.45 (0.95–2.22)	1.83 (0.98–3.42)	0.97 (0.95–0.99)	0.96 (0.59–1.59)
Body mass index (kg/m^2^)						
Low Weight	Reference	Reference	Reference	Reference	Reference	Reference
Normal	1.24 (0.69–2.21)	0.52 (0.12–2.35)	0.95 (0.60–1.49)	0.99 (0.53–1.85)	-	-
Overweight	0.84 (0.47–1.50)	0.72 (0.17–3.09)	0.72 (0.46–1.13)	0.71 (0.40–1.26)	-	-
Obese	0.74 (0.38–1.45)	0.86 (0.27–0.93)	0.79 (0.49–1.26)	0.28 (0.13–0.64)	-	-
Where to go for medical care						
Self-medication, pharmacy, traditional medicine, and other	Reference	Reference	Reference	Reference	Reference	Reference
Health center	1.56 (1.05–2.33)	0.70 (0.34–1.44)	1.35 (1.01–1.80)	2.42 (1.43–4.09)	0.77 (0.32–1.90)	0.77 (0.32–1.90)

## Data Availability

The data supporting the findings of this study are available from the corresponding author upon reasonable request. The data are not publicly available because they contain information that could compromise participant privacy.
